# Supplementary material for: Are carbon emissions trading and green financial instruments synergistic? -Comprehensive quantitative research based on content analysis
Source: PLoS One. 2024 Mar 7;19(3):e0298601. doi: 10.1371/journal.pone.0298601 (PMC10919601; doi:10.1371/journal.pone.0298601)
Supplement: S1 Data — (ZIP) [file pone.0298601.s001.zip › Data/地级市Z_F工作报告.zip!/║╙─╧/╨┬╧τ╩╨/└┤╫╘╣1⁄2╓┌║┼í╛┬φ┐╦╩2╛▌═°í┐_2014.docx]

各位代表：

　　现在，我代表市人民政府，向大会作工作报告，请予审议，并请各位政协委员和特邀列席人士提出意见。

　　一、过去五年工作回顾

　　五年来，本届政府在省委、省政府和市委坚强领导下，在市人大、市政协监督支持下，深入贯彻落实科学发展观，围绕建设幸福新乡和中原经济区强市目标，开拓进取、扎实工作，圆满完成“十一五”规划和市十一届人大一次会议确定的目标任务，奠定了全面建成小康社会的坚实基础。

　　（一）过去五年，科学发展持续加快，综合实力再上新台阶。过去五年是我市发展进程中极不平凡的五年，面对错综复杂的经济形势和竞争激烈的发展环境，我们牢牢把握科学发展第一要务，主动作为，积极应对，克难攻坚，经济社会保持好中趋快的发展态势，综合实力实现新跨越。主要指标晋位升级。2013年完成地区生产总值1766.1亿元，人均生产总值突破3万元，总量是2008年的两倍，由全省第9位上升到第6位，年均增速高于全省1.6个百分点。公共财政预算收入129.5亿元，是2008年的2.7倍，总量由全省第7位上升到第3位，年均增速高于全省2.5个百分点。固定资产投资1561.2亿元，是2008年的2.3倍，总量持续保持在全省前4位。规模以上工业增加值797.9亿元，是2008年的2.2倍。社会消费品零售总额615.2亿元，是2008年的2.2倍。发展质量持续提高。税占比由70.5%提高到73.5%。规模以上工业利税达到268.7亿元，是2008年的2.1倍。产业集聚区对工业增长的贡献率由57%提高到80.2%。高新技术产业增加值比重达到30%，位居全省前列。城镇居民人均可支配收入、农民人均纯收入分别是2008年的1.7倍和1.9倍。城市形象不断提升。先后被授予全国农村改革试验区、全国第一批新能源汽车推广应用城市、中国电池工业之都、全国公交都市建设示范工程创建城市、全国科技进步先进市、国家知识产权示范城市、全国绿化模范城市、全国社会治安综合治理优秀市、中国最佳管理城市等国字号荣誉。

　　（二）过去五年，引资项目强力驱动，发展后劲不断增强。坚持把招商引资作为项目建设的主渠道，把项目建设作为推动发展的综合性抓手。招商引资成效明显。吸引外商直接投资24.87亿美元，市外资金1628亿元。天光科技一期等500个亿元以上引资项目竣工投产，完成投资2000亿元。近百家沿海企业整体转移到我市发展，与深圳安防、LED等产业进入实质谈判，金祥家具产业园等32个产业集群项目落地实施。与30余家央企建立合作关系。在我市投资的世界500强、国内500强企业总数达到57家。新乡海关开关运行，新乡出入境检疫检验机构先期开展工作。完成进出口60亿美元，其中出口38.3亿美元。项目建设扎实推进。实施亿元以上项目1277个，完成投资3126亿元。中原印刷包装产业园等1190个亿元以上项目开工建设，中粮（新乡）现代粮食产业化基地等679个亿元以上项目竣工投产。工业投资占全市固定资产投资比重连续五年超过55%，省市重点项目中高科技项目比重连续5年超过35%。产业集聚发展水平持续提升。产业集聚区建成区面积达到200平方公里，入驻企业4799家，规模以上工业总产值占全市工业的79.2%。高新技术产业开发区、新乡经济技术开发区晋升为国家级园区。在全省6次产业集聚区观摩活动中5次获得豫北片区第1名。5家产业集聚区先后获得省“十强、十快、十先”集聚区称号。

　　（三）过去五年，产业结构不断优化，发展方式加快转变。三次产业结构由14.5：55.8：29.7调整到12：56.6：31.4。新型工业提速发展。六大产业产值达到3339亿元，占全市工业的98%。2013年新兴产业增加值增速高于全市工业9.2个百分点。“车电分离、换电为主”的电动车示范运营模式获得国家有关部门、业界专家和运营商的充分认可，东南牌纯电动轿车允许在全国销售。双鹭医药产业园一期竣工，药品批号转移和二期项目积极推进。河南煤化20万吨乙二醇、银金达PETG薄膜生产线等项目投产。中兵通信科技工业园建成5000台电台产能，与工信部签署中原（物联网）平台协议。亚洲丰隆对新飞增资1.8亿元，并在新乡布局产业园。百威啤酒基地一期20万吨投产，上海宝钢易拉罐项目开工。现代农业稳步发展。大力推进高标准粮田建设，粮食生产实现“十连增”，达到81.2亿斤，被评为全国粮食生产先进市。销售收入10亿元以上农业产业化集群12个，中粮集团等50多家国内外知名龙头企业落户，4899家农民专业合作组织辐射带动46%的农户。市级以上农产品品牌127个，其中国家级品牌45个。全面启动与中国农科院、河南省农科院共建的现代农业示范区建设，农业科技贡献率达到52%。服务业加快发展。服务业对GDP贡献率由18.8%提高到25.2%。完成13家商务中心区和特色商业区规划评审。海宁皮革城等12家国内知名商贸物流企业入驻，宝龙城市广场等大型商业中心开业。建立电子商务产业园，泛亚集团小货郎电子商务平台落户。完成南太行旅游资源整合，旅游业累计接待国内外游客8400万人次，综合收入519亿元。

　　（四）过去五年，新型城镇化科学推进，城乡面貌明显改善。统筹推进中心城市、县城、重点镇和新型农村社区建设，城镇化率提高5.1个百分点。坚持以规划引领城乡建设。把规划作为政府的第一责任、第一资源，推动城市建设由速度型向品质提升型转变。《新乡市城市总体规划》获得国务院批准，基本完成47项专项规划编制评审。中心城区品质提升。新区核心区150个重点项目累计完成投资236亿元，占目标的50%。棚户区和城中村改造项目建成8个，在建65个，拆迁419万平方米，建成308万平方米。启动西部新城规划建设，与中建七局等单位达成初步合作意向。新建和改造城市道路63条、83公里，实施道路渠化改造16处。完成赵定河综合治理和牧野湖等水系景观建设，新增水面1188亩。数字城管实现城乡一体化联网运行。县城组团扩容提质。持续开展“新城杯”竞赛，完成拆迁584万平方米，开工1248万平方米，竣工672万平方米。辉县市县城内涵式发展走在全省前列。平原城乡一体化示范区50平方公里基础设施基本完备，入驻企业60家。镇（乡）区发展步伐加快。用现代城市理念规划建设镇（乡）区，举办两次招商对接活动，签约项目335个，开工220个，亢村镇、孟庄镇等一批小城镇初具规模，5个乡镇跻身2013年全省百强乡镇。积极稳妥推进新型农村社区建设。坚持社区建设与尊重农民意愿、城镇发展、产业发展和促进就业相结合，因地制宜，分类指导，引导镇（乡）区周边社区向乡镇规划区调整靠拢、融合推进。累计投资330亿元，建房3050万平方米，入住农户13.5万户，拆除旧宅6.5万亩、复垦土地3.5万亩。

　　（五）过去五年，改革创新步伐加快，发展活力有效释放。各项改革逐步深化。在全省率先开展“人地挂钩”试点，组织指标交易13次，实现收益13.9亿元。全国农村改革试验区23个试验项目稳步推进，为4.1万户入住社区群众办理城镇户口，为3万户办理房产证。在全省率先实施企业上市引导计划，17家企业进入上市实质阶段，两家在证监会审核。发行全省第一支地市级集合票据和区域集优中小企业集合票据。5次清理行政审批项目，审批项目由663项减少到199项。实施聚能警务战略，基层警力占到市区总警力的70%。乡村两级医疗机构全部实施基本药物制度。市直国有文艺院团体制改革圆满完成。创新能力持续增强。组织实施国家级重大科技专项76项，先后攻克锂电隔膜、复合盾构机等关键技术。组建各类技术研发中心473家，省级以上172家。专利申请量累计达到1.1万件，发明专利占29%。高等院校达到7所，数量位居全省第2位。成立服务高校建设促进地方发展领导小组，创建电池产业技术创新联盟，80%以上的规模以上工业企业与高校和科研院所建立合作关系。质量立市战略深入实施。获得省长质量奖4个。创建省名牌产品63个，数量位居全省第3位。有效注册商标从4366件增加到11268件，中国驰名商标从4件增加到18件，总量均位居全省第3位。成为全国质量强市示范城市争创城市。

　　（六）过去五年，人民生活持续改善，社会大局和谐稳定。财政民生支出达到704.7亿元，占公共财政支出的70.3%。就业创业工作不断加强。累计新增城镇就业63.5万人，下岗失业人员再就业21.9万人，农村劳动力转移就业643.5万人次。在全省率先提出消除零就业家庭并实施动态管理。城镇登记失业率控制在4.5%以内。教育事业加快发展。实施学前教育三年行动计划，新建、改扩建幼儿园231所。开展多元化合作办学，市区153所学校全部参与。改造中小学校舍137.5万平方米，超额完成省定目标。实施职教攻坚计划和全民技能振兴工程，推行“双元制”等职业技能人才培养模式，为企业培养专业技能人才25.6万人。社会保障水平持续提升。县（市、区）全部纳入新农保和城镇居民社会养老保险国家试点，受到国务院表彰。新农合参合率99.5%，创历史新高。在全省率先实现企业养老保险、失业保险市级统筹。“健康快车”共为2812名贫困白内障患者免费实施手术。新建、改扩建农村敬老院40所。累计开工保障性住房855万平方米，竣工445万平方米。生态环境不断改善。新增林地面积37万亩，森林覆盖率达到21.1%，提高3.9个百分点。和谐公园等60处游园绿地相继建成。PM2.5空气自动监测站提前一年投运。县（市）均建成污水、垃圾集中处理工程。集中饮用水源地取水水质达标率保持100%。各项事业协调推进。解决了131万农村人口饮水安全问题。人口自然增长率控制在6.5‰以下。建成农村文化大院1002个。广播电视覆盖率达到99%。驻新部队大力支持地方建设，实现全国双拥模范城“五连冠”。对口支援新疆伊吾县建设成效显著。实行直接联系信访人等工作机制，认真解决群众合理诉求。安全生产形势平稳，民族团结、宗教和谐局面不断巩固，应急管理能力逐步增强，“五五”普法通过国家验收，连续6年被评为全省平安建设先进市，社会大局和谐稳定，各项事业稳步推进。

　　（七）过去五年，政府自身建设不断加强，服务型政府建设取得新成绩。坚持依法行政，自觉接受人大法律监督和政协民主监督，积极听取各民主党派、工商联、无党派人士和人民团体意见，办理市县两级人大代表议案建议3924件，政协委员提案8714件，办结率100%。深入贯彻“两转两提”，开展“三抓两促”活动，修订完善10余项会议制度和议事规则，各级各部门制定完善256个配套制度。对349项重要难点问题进行集中攻坚处理并全部予以解决。建立完善市级领导分包重点工作、重大项目例会等10余项科学管用的工作机制，帮助1000余户企业解决问题2905项，35次银企对接活动共签约贷款1167亿元。落实中央八项规定，2013年市本级“三公”经费同比压减22%；精简文件简报394种；取消各类茶话会、联谊会、团拜会413场（次）。强化行政监察和审计监督，公共资源交易管理中心累计交易项目7577项。

　　各位代表，过去五年的成就来之不易，这是省委、省政府和市委正确领导的结果，得益于历届市四大班子的传承接力，归功于全市人民的辛勤付出，饱含着社会各界的大力支持和帮助。在此，我代表市人民政府，向全市广大干部群众，向驻新官兵和中央、省驻新单位，向在我市创业的投资者、建设者，向所有关心支持新乡发展的各界人士，表示衷心的感谢！

　　各位代表，这五年工作有四点突出体会：一是突出了发展。我们围绕综合实力迈入全省前列目标，把各方力量汇聚到发展上，埋头苦干、奋起直追，综合实力不断晋位升级。二是突出了转变。我们落实主题主线要求，大力发展新兴产业，推动传统产业转型升级，培育优势产业集群，构建了相对合理的产业体系。三是突出了创新。我们坚持把改革创新作为发展的源动力，用创新的思路、机制、举措推动工作、解决难题，有效激发了发展活力。四是突出了实干。我们对确定的目标、认准的事情，一以贯之、毫不动摇地一抓到底，一切立足于实、着眼于效，各项事业全面发展。

　　各位代表，在肯定成绩的同时，我们也清醒地看到存在的问题和不足。主要是：经济总量依然偏小、人均水平较低，全面建成小康社会任务艰巨；工业化、城镇化水平总体不高，服务业发展水平有待提升，资源利用粗放，转变经济发展方式迫切；生态环境污染尚未得到有效治理，雾霾等环境问题还比较严重；县域综合实力不强，改革开放的力度需要进一步加大；公共服务能力不足，就医、就学、就业条件与人民群众需求还有不少差距，城乡居民持续增收难度加大，改善民生任务艰巨；政府职能需要进一步转变，行政效能有待提高。对于这些问题，我们将在今后的工作中努力加以解决。

　　二、今后五年的工作

　　今后五年是我市全面建成小康社会的决战期，是建设幸福新乡和中原经济区强市的关键期。按照中央提出的到2020年全面建成小康社会要求，市委提出提前一年全面建成小康社会，实现生产总值和城乡居民人均收入在2012年基础上翻一番，人均生产总值、城镇居民人均可支配收入、农民人均纯收入达到全国平均水平三个具体目标。我们要按照市第十次党代会部署，不动摇、不折腾、不懈怠，团结一心、继往开来，继续把各项事业推向前进。

　　今后五年政府工作的总体要求是：坚持以邓小平理论、“三个代表”重要思想、科学发展观为指导，深入落实党的十八大和十八届二中、三中全会精神以及省委、省政府和市委决策部署，牢牢把握主题主线，深入推进“一个载体、三个体系”建设，按照“一个总目标、两条工作主线”要求，着力扩投资稳增长，调结构促转型，抓创新增活力，惠民生促和谐，提高发展的质量和效益，加快幸福新乡和中原经济区强市建设进程，为全面建成小康社会奠定更加坚实的基础，为建设“四个河南”、推进“两项建设”做出新乡应有的贡献。

　　经济社会发展的预期目标是：地区生产总值年均增长9—12%；公共财政预算收入年均增长12%左右，全社会固定资产投资年均增长15%，社会消费品零售总额年均增长14%，居民人均可支配收入增长与经济增长同步，保持居民消费价格总体平稳，节能减排完成省定目标任务。

　　今后五年要着力推进以下六个方面的工作：

　　（一）着力扩投资，努力构建加快发展的项目支撑和开放体系。坚持把项目作为加快发展的第一动力，将一切思路、一切战略、一切重点工作项目化，持续实施一批“牵一发动全身”的大项目和“四两拨千斤”的关键项目，完成投资8000亿元以上，利用外商直接投资60亿美元以上。坚持“四集一转”，产业集聚区工业增加值占全市比重超过80%，吸纳就业超过70万人，推动新乡经开区逐步发展为城市新区。推动中原经济区“米”字型快速铁路网在新乡布局，争取京广高铁与晋豫鲁快速铁路在新乡交会，积极推进与郑州轨道交通连接。抓好新乡国际陆港和综合保税区前期工作，争取建设国家铁路二类口岸，打造中原经济区开放高地。

　　（二）着力调结构，努力构建更具竞争优势的现代产业体系。按照“做强工业、做大服务业、做优农业”要求，推动产业向高端化、终端化、高效益方向发展。坚定不移地推动新型电池与电动车、生物与新医药、电子信息、现代煤化工等新兴产业发展壮大，改造提升传统优势产业，努力再造一个全新的工业体系。巩固农业基础地位，初步形成以粮食增产、产业化带动、品牌运作、科技支撑为主导的现代农业发展体系。加快旅游、金融、物流、电子商务等行业发展，初步形成与新型工业化、新型城镇化、农业现代化、信息化互动的现代服务业体系。

　　（三）着力促统筹，努力构建以人为本的新型城镇和统筹城乡发展体系。坚持“以人为本、优化布局、生态文明、传承文化、产城互动、科学有序”原则，按照“核心带动、轴带发展、节点提升、对接周边”要求，推动中心城市组团式发展、县城和重点镇内涵式发展、新型农村社区集聚式发展，打造城乡一体、功能完善、生态宜居的现代田园城市。三年内基本完成棚户区和城中村改造，推动辉县市、卫辉市迈入中等城市行列，推动平原城乡一体化示范区率先建成全省城乡一体化先行区。把推进农业转移人口市民化作为首要任务，推动城镇常住人口率先实现城镇基本公共服务全覆盖。

　　（四）着力求提升，努力构建创新驱动发展的科教支撑体系。强化科技引领发展的理念，注重发挥企业家才能，加强产品创新、品牌创新、产业组织创新、商业模式创新，不断增强创新发展驱动力。深入实施科技创新十大工程，加大企业研发中心建设力度，抓好重大科技专项实施，协同创建郑洛新自主创新综合试验区，力争2015年成为国家创新型试点城市。支持驻新高校和科研院所发展，促进更多成果在新乡转化推广。营造崇尚创新、追求创新的氛围，增强全民创新创造活力。

　　（五）着力促协调，努力构建美丽新乡的生态文明保障体系。良好的生态是最公平的公共产品，我们决不以牺牲环境为代价换取一时的发展。要扎实推进“蓝天工程”、“碧水工程”和“乡村清洁工程”实施，加快生态水系、生态廊道建设，抓好雾霾、水污染、农村环境等重点难点问题治理，健全生态环境保护责任追究和环境损害赔偿制度，努力为群众创造更舒适的居住生活环境。推动德润新乡建设，争创全国文明城市，提升全民整体素质，使新乡先进群体薪火相传，永葆活力，打造道德文明高地。

　　（六）着力惠民生，努力构建幸福新乡的民生保障和公共服务体系。把人民群众对美好生活的向往，作为我们的奋斗目标。新增财力60%以上用于民生支出，每年办成一批群众期盼的实事，城乡居民收入基本实现翻番。城乡居民基本社会养老保险参保覆盖率保持在99%。贫困人口减少23万以上。以保障权利公平、机会公平、规则公平为核心，健全政务公开制度，深化行政审批制度改革，规范行政行为，坚持依法行政，完善提升“四位一体”服务监督体系，打造行为规范、运转协调、公正透明、廉洁高效的政务环境。

　　各位代表，全面建成小康社会、实现综合实力迈入全省前列、为全省发展做出更大贡献，是省委、省政府的殷切期盼，是全市人民的共同心愿，是历史赋予我们的光荣使命！梦想孕育着奇迹，事业出自于艰辛。尽管挑战众多，任务艰巨，但只要我们用“钉钉子”的精神和抓铁有痕、踏石留印的劲头，一锤一锤接着敲，一件一件干到底，就一定能够在现有基础上，顺势而为、乘势而上，推动各项事业实现新的更大发展！

　　三、2014年工作安排

　　今年是新一届政府开局之年，要全面贯彻落实上级和市委决策部署，把改革创新贯穿经济社会发展各领域和环节，按照“调中求进、变中取胜、转中促好、改中激活”要求，狠抓重点工作落实，推动经济社会发展稳中有进、稳中有为、稳中向好，为五年工作开好头、起好步。主要预期目标是：地区生产总值增长9.5%以上，公共财政预算收入增长12%，全社会固定资产投资增长20%，社会消费品零售总额增长14%，城镇登记失业率控制在4.5%以内，居民人均可支配收入与地区生产总值增长基本同步，完成省定节能减排目标。

　　今年重点做好以下工作：

　　（一）深入推进招商引资和项目建设。加大开放招商力度。突出重点区域和重点行业，强力推进产业集群、延链补链和央企招商。积极促成深圳安防、LED、电子信息等产业向我市集群式转移；推动以天光科技为龙头的手机配套、以百威啤酒为龙头的食品加工等产业链招商；推进中国电科重组电池产业、中国建材整合水泥产业、中航专用汽车、中原健康产业园等央企合作项目取得实质成果。实际利用外资增长10%，市外资金实际到位增长15%，新签约亿元以上项目200个。强力推进重点项目建设。实施5000万元以上项目1075个，完成投资1077亿元，其中亿元以上项目864个。加快光电信息产业园等580个在建项目实施进度，推进百威啤酒二期等350个重点项目开工建设；确保日升数控装备工业园、香港长德商贸物流城一期等204个重大项目竣工投产。做好华电2×100万千瓦超超临界发电机组等143个重点谋划项目的前期工作。提升产业集聚区发展水平。继续实施产业集聚区“1＋2＋3”工程，全力引进实施一批重大产业项目。省级产业集聚区主营业务收入超过2000亿元，增长15%；固定资产投资850亿元，增长22%。新乡高新区、新乡经开区要用好国家级园区金字招牌，努力打造千亿园区。

　　（二）加快培育新型工业体系。新型电池与电动车。抓好电动车示范运营，力争通过国家中期考核。加快河南锂动1.2亿安时动力锂电、中科6亿平方米锂电隔膜等项目实施。加大科隆集团新能源专用车产业化基地、中航集团汽车转向器等项目推进力度，提升产业配套能力。生物与新医药。抓好中原经济区生物医药产业园、双鹭生物产业园两个基地型项目建设，争取华兰生物与丹麦CMC公司合作项目尽快实施。加快华星药厂与军事科学院合作开发重组肝活素、佐今明大健康产业园等项目建设。现代煤化工。抓好达新源年产3.6万吨PETG薄膜连续化生产线、河南现代包装5万吨BOPP、晋开延化年产60万吨合成氨80万吨尿素、心连心120万吨复合肥等项目建设。新开工白鹭化纤4万吨氨纶项目，力争3年内达到10万吨产能。制冷及电子信息。以新飞电器等企业为重点，完善产业链条，提高本地配套率，加快新产品研发，进一步壮大制冷产业。推进天光科技二期、索泰克光电二期等项目建设，提升手机配套产业发展水平。加快中原（物联网）平台建设，大力发展智慧产业。加大中兵通信科技工业园、中国电波科技城等重点项目实施力度。推进3G、4G等信息基础设施建设，打造无线城市、智慧城市。特色装备制造。以装备集成为突破口，壮大起重机械和振动机械两大优势产业集群，推进豫飞重工船用及轨道式集装箱起重机、平原航空机电设备园区等70个项目建设。传统优势产业。力争上海宝钢易拉罐项目一期10月份投产，绿生堂加多宝加工基地上半年投产，广药集团王老吉项目开工。加快酒鬼酒2万吨白酒生产线、美日康15万吨树莓深加工等项目实施，推动食品产业升级。建设纺织服装产业园，推动开元纺织年产1200万件高档针织服装等8个项目入驻。推进造纸、水泥行业横向联合，淘汰落后产能，压缩过剩产能。

　　（三）提升现代农业发展水平。提高粮食综合生产能力。加强引黄调蓄、小农水重点县等农田水利建设，扩大引黄灌溉面积。大力实施高标准粮田“百千万”工程，新增高标准粮田60万亩，粮食产量稳定在81亿斤以上。发展品牌农业。培育年销售收入10亿元以上产业化集群10个，做大做强“中国第一麦”、“中国第一米”、封丘金银花等品牌。优化农业结构，新建100个标准化规模养殖场。积极发展都市生态农业，保障“菜篮子”产品生产供应，经济作物面积稳定在350万亩、总产420万吨以上。加快农业科技成果转化。推进现代农业示范区建设，抓好农业科技研发、高效农业生产等十大工程实施，打造全国重要的农业科技研发、示范推广基地。构建新型农业经营体系。推进体制机制创新，鼓励承包经营权向专业大户、家庭农场、合作社、农业企业创新流转，新增土地流转面积25万亩，新发展农民合作社500家。培养有文化、懂技术、会经营的新型职业农民，推动农民创业就业。加大“新网工程”建设力度，构筑农村流通服务新网络。

　　（四）推进现代服务业发展壮大。实施服务业提升工程，加快物流、金融、旅游、文化、科技研发、医疗健康等十大行业发展。现代物流业。完成现代物流业发展规划修编，出台促进物流业健康发展的意见。抓好小店、南环等5大综合物流园区规划建设；加快中部医药物流城、中粮粮食物流园等36个重大物流项目实施进度。金融服务业。强化银企对接工作机制，新增贷款120亿元以上。做好2家已报会在审企业跟踪服务，推进科隆新能源进入辅导程序，力争1-2家企业在“新三板”挂牌。积极组建农村商业银行和村镇银行。做好1.2亿元区域集优集合票据发行工作。旅游业。培育南太行山水度假旅游、牧野文化体验旅游、沿黄乡村游憩旅游等3大旅游产业集群，旅游总收入达到175亿元，增长15%。提升南太行景区发展水平，加快景区游客服务中心和道路建设，旅游销售收入增长20%，3年内整体争创5A级景区。电子商务。培育电子商务、网络营销等新型流通业态，开工建设泛亚集团1亿港元小货郎电子商务平台，建立大学生电子商务创业孵化园。

（五）科学推进新型城镇体系建设和统筹城乡发展。加快农业转移人口市民化。强化“一基本两牵动”，按照“进得来、落得住、转得出”要求，加快制定促进农业转移人口进城落户具体办法，把促进有能力在城镇稳定就业和生活的常住人口有序实现市民化作为首要任务，稳步推进城镇基本公共服务常住人口全覆盖。进一步强化规划在城镇化中的基础和统领作用。全面深化并执行好60项专项规划，认真梳理中心城区控规，制定科学化、系统化、规范化的实施办法，作为指导城市建设重要依据。编制全域新乡现代城镇体系规划，构建可持续发展现代城镇体系。深入推进数字城管城乡一体化，再夺全省城市精细化管理优秀城市称号。继续提升中心城区品质和承载力。抓好新区核心区150个重点项目建设，完成投资50亿元以上。加快推进棚户区和城中村改造，棚户区完成拆迁48万平方米，新开工10个项目、7136套，基本建成5025套。新开工19个城中村改造项目，完成拆迁190万平方米，建设安置房86万平方米。以“一场两路三片区”为重点，推动西部新城完成投资20亿元以上，尽快启动直升机场迁建工程。加快中心商务区和特色商业区建设，推动老城区商业资源合理疏散、多中心布局。开工宏力大道东段等6条城市道路，启动高铁站区域基础设施二期工程建设，完成8处重要交通路口渠化改造。抢抓卫河申遗机遇，加快实施卫河生态文化项目，完成市区段6.8公里清淤工程；做好东、西孟姜女河综合治理前期工作；完成南护市沟疏浚，促进市区渠系整体贯通。完成南水北调配套工程及调蓄工程建设。推动县城和重点镇内涵式发展。把县城作为农村人口转移主阵地，加快旧商业街区和城中村改造，完成拆迁300万平方米，开工200万平方米。按照一二三产业复合和经济、生态、人居功能复合的理念，加快平原城乡一体化示范区发展。提高镇（乡）区发展建设质量，推动唐庄镇、小冀镇等20个中心镇逐步成为各具特色的小城镇。推动新型农村社区集聚式发展。按照“分类指导、科学规划、群众自愿、就业为本、量力而行、尽力而为”要求，抓好区位优势明显、群众入住社区愿望高、建设规模大、基础设施和公共服务设施配套基本到位的一类社区建设。促进新型农村社区与镇（乡）区、产业集聚区发展建设有机结合。新开工建房面积700万平方米，入住2万户，拆除旧宅1.7万亩，复垦或恢复生态5000亩。推进“美丽乡村”建设，逐步打造一批富有特色的示范村。构建内联外畅的综合交通体系。加快公交都市建设，建成市政府至森林公园至市政府快速公交环线，规划建设段村等4个综合场站，新增新能源公交车100辆。加快实施鹤辉高速、G107马村立交至黄河大堤段改建等项目。完成新乡境内快速铁路布局规划、郑州至济南快速铁路郑州新乡段预可研报告，适时启动郑州至济南快速铁路新乡濮阳段、新乡至焦作快速铁路等项目的前期工作。

　　（六）大力实施科教强新战略。积极推进国家创新型试点城市创建。深入推进战略新兴产业培育等十大创新工程，高新技术产业增加值增长25%。实施发明专利倍增计划，专利申请量保持在3000件以上，发明专利20%以上，实施科技成果转化项目20项，确保通过年度考核。加强创新能力建设。组织实施重大科技专项12项，申报省级以上科技计划项目40项，推动广瑞公司、天丰集团申报国家级企业技术中心，天光科技等5家企业申报省级高新技术企业，新组建40家市级企业研发中心。加快重点职教园区建设，深入推进“双元制”培训，完成高技能人才培养6000人。大力推进协同创新。推动85%的规模以上企业与科研院所、高校建立长期稳定合作关系，在生物医药等领域组建产业技术创新联盟，建设一批协同创新中心。创新人才发展机制，引导市内外高素质人才在我市就地转化成果、领办创办企业，促进更多创新资源和创新要素向我市集聚，更多创新成果在我市转化为现实生产力。优化创新环境。充分发挥政府的组织员、保育员和服务员作用，鼓励支持科技创新、制度创新和管理创新。制定推进科技金融创新实施意见，构建多元科技投融资体系，推动全社会研发投入比重达到1.9%。

　　（七）切实保障和改善民生。继续加大民生和社会事业投入力度，强化财政资金使用监管，全面完成省市承诺的实事办理工作。继续推进创业型城市创建。深入实施创业政策扶持、创业培训等十大创建工程，加快大学生创业孵化园建设，支持高校毕业生等群体创业就业，城镇新增就业7.3万人，失业人员再就业2.27万人。稳步推进企业工资集体协商和集体合同制度，逐步完善仲裁与劳动监察、劳动关系协作机制，启动整治拖欠进城务工人员工资三年行动计划，构建和谐劳动关系。提升社会保障水平。扩大城乡居民社会养老保险覆盖范围，城镇职工基本养老保险参保总数达到83.2万人，失业保险参保总数达到44.3万人，城镇基本医疗保险参保总数达到142.7万人，养老金按时足额发放和社会化发放率100%，新农合参合率保持在99%以上。健全养老服务体系，每千名老年人拥有养老床位数27张以上。抓好公共租赁住房和廉租住房并轨运行，建设公共租赁住房30281套，其中续建26525套，新开工3756套，基本建成8748套，分配入住6033套。促进教育均衡发展。年内基本实现县域内多元化合作办学全覆盖，到2015年基本取消择校。推进农村义务教育薄弱学校改造、农村初中改造二期工程等建设，改造校舍4.8万平方米。提高医疗服务保障水平。加快市中心医院东区医院、市一院全科医生临床培养基地等项目建设，推进县级医院、中心乡镇卫生院、村卫生室标准化建设，为困难群众减免医疗费用1000万元以上。强力推进生态环境保护。精心呵护我们的城市，是各级政府、各个企业和全市人民的共同责任，我们都要进一步行动起来，保护好我们赖以生存的家园。要加大雾霾天气整治力度，拆除改造集中供热范围内10蒸吨以下燃煤小锅炉，完成所有燃煤电厂运行脱硫、脱硝设施，启动餐饮业油烟整治，推广电动公交车和电动汽车，减少机动车污染排放，大力开展建筑工地粉尘综合治理。加快污水处理厂建设，抓好水污染防治，确保河流水质达标和饮用水源地水质安全。积极开展农村连片综合整治，抓好畜禽养殖污染治理，合理规划推进村头、地边的排污企业升级改造并向园区集聚。完善提升中心城市、县城、产业集聚区、重点镇之间的生态隔离工程，完成造林10万亩。加大扶贫开发力度。抓住省实施“三山一滩”群众脱贫工程机遇，加快封丘县李庄乡试点整体搬迁工作；完成260户、1200人深石山区贫困农户搬迁扶贫任务；再实现4.6万农村贫困人口稳定脱贫。加强食品药品安全监管。健全食品药品安全保障和监管体系，构建从田间到餐桌全过程的监管制度和机制，推进食品安全放心主食工程，食品抽检不少于6000批次，60%的大中型餐饮服务单位达到“透明厨房”标准；开展药品安全专项整治，基本药物抽验覆盖率达到100%，质量监督抽验合格率98%以上。强化监管责任和责任追究，严查制假售假行为。促进社会事业均衡发展。再解决20万农村居民的饮水安全问题。新发展农村有线电视用户2.5万户。县（市、区）全民健身活动中心覆盖率达到30%以上。大力发展文化事业，组织好2014年海峡两岸传统文化与现代化研讨会。加强国防动员和后备力量建设，积极做好驻新部队服务保障，促进军民深度融合发展。稳定低生育水平，人口自然增长率控制在6.5‰以内。推动防震减灾、统计、史志、外事、侨务、档案、气象、测绘、人防等工作均衡发展。加强和创新社会治理。抓好社会矛盾隐患排查和风险评估，强化应急管理。深入实施聚能警务战略，加强社会治安综合治理，严厉打击违法犯罪活动。加强和改进信访工作，依法依规解决群众反映的问题。加大“六五”普法工作力度，做好法律援助工作。牢固树立安全发展理念，强化安全创建，深化重点行业和领域整治，从严从重打击非法违法和违规违章生产行为，遏制事故发生，维护社会大局和谐稳定。

　　（八）全面深化各项改革。按照中央和省统一部署，制定出台我市全面深化改革实施意见，推进一批重点领域和关键环节的改革，争取一些改革工作成为全省乃至全国试点。深化行政管理体制改革。按照“能放则放”的原则，在现有199项行政审批事项基础上，进一步抓好减少、整合、下放工作，最大限度整合职能、理顺关系。推动行政管理权限从县级政府向经济发达镇转移。推进现代市场发展体系改革。围绕以管资本为主加强国有企业监管，研究探索新形势下推动国有企业改革发展工作。深化财税体制改革，建立行之有效的全过程预算绩效管理机制。加强政府债务管理，有效防范和化解财政风险。按照国家、省统一部署，推进水、石油、天然气、电力、电信等领域价格改革。抓好其他领域改革。稳步推进全国农村改革试验区工作，加快实施城乡户籍管理等10项改革试验任务，全面完成13个已建成社区产权制度改革，有序推进具备条件的建成社区产权制度改革。深入实施人地挂钩试点，逐步建立城乡统一的建设用地市场，实施节余建设用地指标及补充耕地指标交易5千亩，实现收益6.5亿元。

　　四、努力建设人民满意的服务型政府

　　认真贯彻党中央、国务院和省委、省政府关于地方政府职能转变和机构改革要求，把提升政府的治理能力和工作水平与开展党的群众路线教育实践活动有机结合，深入推进“两转两提”，进一步改进作风、转变职能、提高效率、优化服务。

　　（一）我们要切实转变作风。始终把人民满意作为第一标准，扎实开展第二批党的群众路线教育实践活动，深入落实中央八项规定和省委、市委有关要求，持续解决“四风”突出问题，健全改进作风常态化制度。要厉行节约，严控“三公”经费等一般性支出，公用经费定额压减5%，在政府过紧日子中让人民群众过上好日子。要深入群众、深入一线，勇于担当、敢于负责，对重点工作一竿子插到底，不达目的不罢休、不获全胜不收兵。要健全完善重点工作责任体系，加强督促检查和行政效能监察，严格行政问责，奖优罚劣，确保重大决策部署落到实处。

　　（二）我们要加快转变职能。围绕市场在资源配置中起决定性作用和更好发挥政府作用，深入推进职能转变和机构改革，强化政府公共服务、市场监管、社会治理、环境保护等职责，推动政府职能加快向创造良好发展环境、提供优质公共服务、维护社会公平正义转变。严控机构编制，确保财政供养人员只减不增。落实统一的市场准入制度，试行负面清单管理模式。继续清理行政审批事项和具有审批性质的管理事项，对保留的审批事项实行目录管理、简化手续、优化流程、逐步公开，加强政务服务体系建设，切实提高行政效能。

　　（三）我们要坚持依法行政。严格按照法定权限和程序行使权力、履行职责，推进政府工作法治化。自觉接受市人大的法律和工作监督、市政协的民主监督、新闻舆论监督和社会公众监督，认真听取人民群众和社会各界的意见建议，同心同德、团结一致做好工作。深入推进服务型行政执法，强化行政执法责任和过错追究。深化政务公开，让人民监督权力，让权力在阳光下运行。实行重大决策和政府合同集体会商与专家论证，确保群众的知情权、参与权、表达权和监督权。

　　（四）我们要自觉廉洁从政。认真落实党风廉政建设责任制和领导干部廉洁自律的各项规定，完善提升公共资源交易中心等“四位一体”服务监督体系，规范土地出让、资源开发、项目招标、行政审批、政府采购、资金安排、重大工程建设等管理，探索从源头和机制上防止腐败的有效措施。完善行政监察、审计监督等内部监督机制，纠正部门和行业不正之风。全市公务员都要按照习近平总书记要求，既严以修身、严以用权、严以律己，又谋事要实、创业要实、做人要实，常思贪欲之害、常怀律己之心，真正做到为人民服务，对人民负责，受人民监督，让人民满意。

　　各位代表！建设中原经济区强市的美好前景鼓舞着我们，人民群众过上更加美好生活的热切期望鞭策着我们。让我们紧密团结在以习近平同志为总书记的党中央周围，在省委、省政府和市委坚强领导下，齐心协力，砥砺奋进，开拓创新，共创新乡的美好明天，为建设“四个河南”，实现中华民族伟大复兴的中国梦做出我们的贡献！
